# Safety and efficacy of extracranial vertebral artery stenting in elderly patients with symptomatic vertebral artery stenosis

**DOI:** 10.3389/fnagi.2025.1667157

**Published:** 2025-12-10

**Authors:** Miao Wu, Zhiyong Zhang

**Affiliations:** Department of Neurology, Beijing Geriatric Hospital, Beijing, China

**Keywords:** vertebral artery stenosis, elderly, stroke, in-stent restenosis, atherosclerosis

## Abstract

**Objectives:**

Symptomatic stenosis of the extracranial vertebral artery is an important cause of posterior circulation stroke. Endovascular stenting has shown promise for these lesions, but evidence in patients aged ≥ 70 years is limited. This study assessed perioperative safety and intermediate-term efficacy of extracranial vertebral artery stenting in elderly patients (≥70 years) by comparing rates of complications, restenosis, stroke, and death with those in younger patients.

**Methods:**

We retrospectively analyzed patients with symptomatic extracranial vertebral artery stenosis (VAS) treated at our center from 2019 to 2024. Patients were divided into two groups (≥70 vs. <70 years). All patients had failed medical therapy (antiplatelet ± statin) and underwent stent placement. We compared perioperative adverse events, in-stent restenosis (ISR), target-vessel stroke, and any stroke or death between groups.

**Results:**

Among 224 patients (93 aged ≥ 70, 131 aged < 70), technical success was 100%. Median age was 74 (IQR 72–77) in the ≥70 group and 63 (IQR 59–66) in the <70 group. Perioperative complication rates did not differ significantly (4.3% vs. 3.1%; *p* = 0.721). Over a mean follow-up of 15 months, ISR occurred in 13.5% of ≥70 patients and 17.6% of <70 patients (*p* = 0.50), and target-vessel stroke occurred in 3.3% vs. 1.6% (*p* > 0.05). Multivariate analysis showed that bare-metal stent use and hyperuricemia independently predicted ISR. However, the combined outcome of any stroke or death was significantly higher in the ≥70 group than in the <70 group (14.6% vs. 3.2%, log-rank *p* < 0.05), which was largely attributed to more severe atherosclerosis and a higher burden of comorbidities in the older population.

**Conclusion:**

In symptomatic patients with extracranial VAS, endovascular stenting in the elderly (≥70 years) appears comparably safe to younger patients, with similarly low restenosis and target-stroke rates. Use of bare-metal stents and hyperuricemia were associated with higher ISR. These findings support considering vertebral stenting in elderly patients, but emphasize the need for close follow-up.

## 1 Introduction

Posterior circulation strokes tend to have higher recurrence and mortality rates than anterior circulation strokes. Atherosclerosis of the proximal vertebral artery (V1 segment) is a common cause of posterior ischemic events. In the New England Medical Center Posterior Circulation Registry, 20% of patients with posterior ischemic stroke had V1 vertebral artery lesions (Wityk et al., 1998). Standard medical therapy (antiplatelet agents and vascular risk factor management) is effective for many patients, but those with severe or recurrent stenosis remain at high risk of stroke. Endovascular stenting of extracranial VAS is an attractive option: most studies have demonstrated high technical success and low perioperative risk, with low long-term restenosis and stroke rates (Jenkins and Stewart, 2017). Despite this, the safety and efficacy of vertebral artery stenting in very elderly patients (≥70 years) has not been well studied. Given the aging population and higher prevalence of atherosclerosis in older adults, understanding outcomes in this subgroup is important. We therefore performed a retrospective cohort analysis comparing perioperative and mid-term outcomes of VA stenting in patients aged ≥ 70 years versus those <70 years, to provide evidence for individualized treatment strategies in elderly patients.

## 2 Materials and methods

### 2.1 Study population

This retrospective one-center study was approved by the ethics committee of Beijing Geriatric Hospital (Approval No. BJLNYY-2022-013), and informed consent was provided by the participants to participate. We reviewed medical records of all patients who underwent endovascular stenting for symptomatic extracranial VAS at Beijing Geriatric Hospital from January 2019 to September 2024. Inclusion criteria were: (1) Digital subtraction angiography (DSA) confirmation of ≥70% stenosis at the origin or V1 segment of a vertebral artery; (2) treatment with stent placement and at least 3 months of angiographic or ultrasound follow-up; (3) prior medical therapy with antiplatelets (single or dual antiplatelet agents) therapy for vascular risk control, yet persisting posterior circulation ischemic symptoms (dizziness, syncope, transient ischemic attack, or stroke) (Ortega-Gutierrez et al., 2019). Exclusion criteria included: non-atherosclerotic stenosis (e.g., dissection, fibromuscular dysplasia), concurrent intracranial aneurysm or AV malformation, prior surgery on the ipsilateral vertebral artery, or allergy to iodinated contrast. Symptomatic stroke was defined by acute positive diffusion-weighted MRI (DWI) or subacute FLAIR changes in the posterior circulation.

### 2.2 Endovascular procedure

The procedures were performed under local anesthesia via either the radial or femoral artery approach. Intravenous heparin was administered intraoperatively to maintain an activated clotting time (ACT) between 250 and 300 s. All patients underwent aortic arch and cerebral angiography to quantitatively assess the degree and length of the stenotic lesions. Endovascular revascularization was subsequently performed based on angiographic findings. The drug-eluting stent(DES) used in this study included Maurora stent (Alain Medical, Beijing, China) and Bridge stent (Shanghai Microport NeruoTech, Shanghai, China). Bare metal stents (BMS) included Carbofilm coated stent (Alvi Medical, Italy), Trump stent and Apollo stent (MicroPort Medical, Shanghai, China). For lesions with severe stenosis (<2 mm in diameter) that impeded stent delivery, pre-dilation was performed using the Secticide balloon (Achieva Medica, Suzhou, China). Under micro-guidewire guidance, the stent was advanced to the stenotic segment of the vertebral artery (V1 segment) and deployed precisely after proper positioning. Distal protection devices were not used. Final angiography confirmed a residual stenosis of less than 15%, with good patency of both the stent and distal vessels. The catheter system was then withdrawn. All procedures were performed by experienced interventional physicians.

### 2.3 Periprocedural medication

Pre-procedure, all patients received aspirin (100 mg/day) and clopidogrel (75 mg/day) for at least 3 days. A 300 mg clopidogrel loading dose was given if antiplatelet therapy had not been initiated. Post-stenting, dual antiplatelet therapy was continued for at least 6 months, followed by lifelong aspirin.

### 2.4 Parameters assessed and follow-up

Technical success was defined as residual stenosis <15% after stent deployment. Standardized follow-up was scheduled at 1, 3, 6, and 12 months post-procedure, then annually, including: neurological examination, carotid duplex ultrasound, laboratory tests. ISR was defined as >50% luminal narrowing within the stented segment as detected by follow-up vascular imaging (either CT angiography or DSA), or based on ultrasound diagnostic criteria: peak systolic velocity (PSV) > 170 cm/s, end-diastolic velocity (EDV) > 45 cm/s, and a PSV ratio ≥ 2.7 (Li et al., 2020). Any new neurological symptoms triggered brain MRI. Data on perioperative complications (stroke, death, major bleeding within 30 days) and long-term outcomes were systematically collected.

### 2.5 Statistical analysis

Survival analysis was performed using the Kaplan-Meier method with between-group comparisons assessed by log-rank test. Potential predictors of ISR were first evaluated through univariate analysis, with statistically significant variables (*P* < 0.05) subsequently incorporated into multivariate Cox proportional hazards regression models to identify independent risk factors. All statistical analyses were conducted using IBM SPSS Statistics (version 29.0, Armonk, NY).

## 3 Results

### 3.1 Patient and baseline characteristics

A total of 224 patients were enrolled in this study. The ≥70-year-old group comprised 93 patients [median age 74 (IQR 72–77) years], including 4 who underwent bilateral vertebral artery stenting (total 97 vessels treated). The <70-year-old group included 131 patients [median age 63 (IQR 59–66) years], with five receiving bilateral stenting (total 136 vessels treated). Technical success was achieved in 100% of procedures across both groups. Baseline demographic and clinical characteristics did not differ significantly between groups (Table 1).

**TABLE 1:** Baseline characteristics comparison between groups.

Parameters assessed	<70 years (*N* = 131)	≥70 years (*N* = 93)	*P*-value
**Demographics**			
Age (years)	63 (59–66)	74 (72–77)	<0.001
Male sex	103 (78.6%)	64 (68.8%)	0.097
**Symptoms**			
Dizziness	95 (72.5%)	66 (71.0%)	0.799
Syncope	2 (1.5%)	5 (5.4%)	0.130
Posterior circulation infarction/TIA	34 (26.0%)	22 (23.7%)	0.695
**Comorbidities**			
Hypertension	107 (81.1%)	75 (80.6%)	0.845
Diabetes mellitus	69 (52.7%)	57 (61.3%)	0.200
Hyperlipidemia	77 (58.8%)	51 (54.8%)	0.557
Hyperhomocysteinemia	63 (48.1%)	49 (52.7%)	0.498
Hyperuricemia	19 (14.5%)	8 (8.6%)	0.181
Renal insufficiency	6 (4.6%)	8 (8.6%)	0.267
Heart disease	41 (29.7%)	34 (36.6%)	0.276
- Coronary artery disease	38 (29.0%)	31 (33.3%)	0.490
- Atrial fibrillation	3 (2.3%)	3 (3.2%)	0.694
Smoking	52 (39.7%)	33 (35.5%)	0.522
**Procedure details**			
**Surgical side**			
- Left	56 (42.7%)	36 (38.7%)	0.635
- Right	70 (53.4%)	53 (57.0%)	0.598
- Bilateral	5 (3.8%)	4 (4.3%)	1.000
**Approach**			
- Radial artery	17 (13.0%)	20 (21.5%)	0.09
- Femoral artery	114 (87.0%)	73 (78.5%)	
Vertebral artery stents	136	97	
- DES	104 (76.5%)	83 (85.6%)	0.086
- BMS	32 (23.5%)	14 (14.4%)	
Stent diameter (mm)	4 (3.5–4.5)	4 (3.75–4.5)	0.595
Stent length (mm)	13 (12–13)	12 (12–13)	0.871
Balloon pre-dilation (yes)	25 (18.4%)	12 (12.4%)	0.216
Post-dilation pressure (atm)	12 (12–14)	12 (11–14)	0.493
Preoperative stenosis (%)	80 (70–85)	75 (72.5–82.5)	0.510

Count data are reported as% (*n*). Continuous data are reported as median (IQR). DES, drug-eluting stent; BMS, bare-metal stent; TIA, transient ischemic attack.

### 3.2 Periprocedural outcomes

The 30-day complication rate was 4.3% (4/93) in the ≥70-year-old group and 3.1% (4/131) in the <70-year-old group (*P* = 0.72). In the elderly group, complications included three cases of new-onset stroke—one of which was a fatal cerebral infarction—and one case of transient hypotensive response following radial artery puncture. No major bleeding events occurred in this group. In the younger group, there were two cases of new-onset stroke and two cases of major bleeding (one gastrointestinal and one genitourinary), with no reported deaths. There were no statistically significant differences in the incidence of individual adverse events between the two groups (Table 2), suggesting that periprocedural safety was comparable in patients aged ≥ 70 years.

**TABLE 2:** Comparison of perioperative adverse events.

Adverse event	<70 years (*N* = 131)	≥70 years (*N* = 93)	*P*-value
Overall complications	4 (3.1%)	4 (4.3%)	0.721
New-onset stroke	2 (1.5%)	3 (3.2%)	0.651
Major organ hemorrhage	2 (1.5%)	0 (0.0%)	0.512
Procedure-related mortality	0 (0.0%)	1 (1.1%)	0.415

Count data are reported as% (*n*).

### 3.3 Follow-up outcomes

**FIGURE 1:**
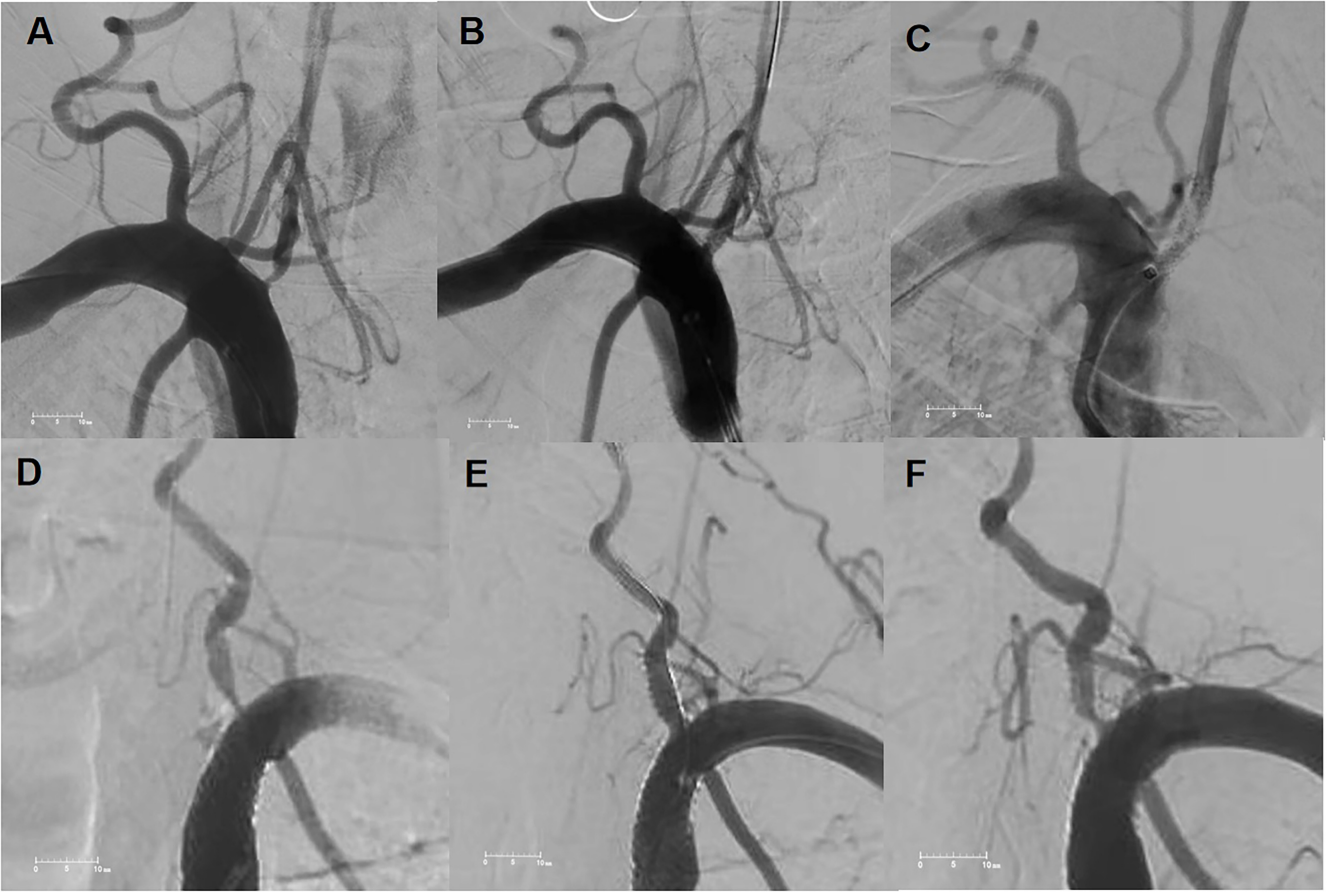
(A) Preprocedural angiography showing severe stenosis of the right vertebral artery. (B) Immediate post-stenting angiogram revealing a widely patent lumen with residual stenosis < 5%. (C) Follow-up angiography at 9 months demonstrating significant in-stent restenosis (ISR) (>50% diameter stenosis) in the right vertebral artery. (D) Preprocedural angiography showing severe stenosis of the left vertebral artery. (E) Immediate post-stenting angiogram demonstrating adequate lumen restoration with residual stenosis < 10%. (F) Follow-up angiography at 12 months showing severe ISR (> 70% diameter stenosis) in the left vertebral artery.

After excluding one perioperative death and nine patients lost to follow-up (total exclusions: 10 patients/10 vessels; six from the <70-year cohort and four from the ≥70-year cohort), the remaining 214 patients (100% follow-up compliance) completed a minimum of 3 months of follow-up. The mean follow-up duration was 15 months in the <70-year group and 14 months in the ≥70-year group. ISR is a common complication following vertebral artery stenting (Figure 1). ISR was observed in 22 patients (17.6%) in the younger cohort and 12 patients (13.5%) in the elderly cohort (*P* = 0.500), indicating no significant difference between groups. Univariate analysis identified the use of BMS, hyperuricemia, and balloon inflation pressure as potential predictors of ISR. Multivariate Cox regression analysis further confirmed that BMS implantation [hazard ratio (HR) 3.334; 95% confidence interval (CI), 1.594–6.974] and hyperuricemia (HR 2.899; 95% CI, 1.189–7.067) were independent risk factors for ISR (Table 3).

**TABLE 3:** Cox regression analysis showing factors associated with in-stent restenosis (ISR).

Risk factor	Hazard ratio	95% Confidence interval		*P*-value
Balloon dilation pressure	0.919	0.781	1.082	0.312
BMS	3.334	1.594	6.974	0.001
Hyperuricemia	2.899	1.189	7.067	0.019

BMS, bare-metal stent.

**FIGURE 2:**
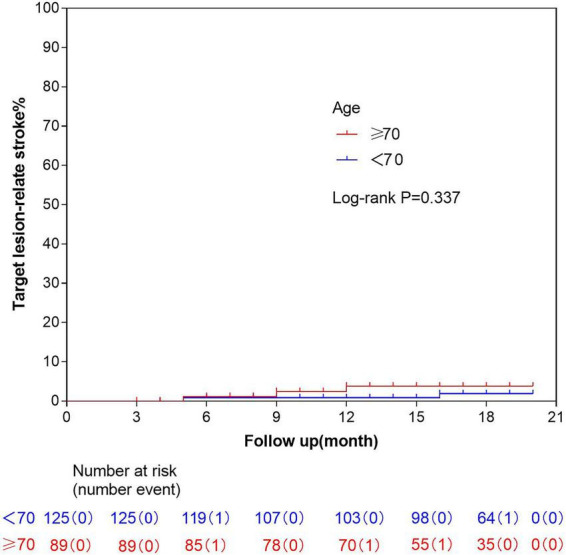
Kaplan-Meier curve of target lesion-relate stroke.

During the follow-up period, a total of two patients (1.6%) in the <70-year-old group experienced target vessel territory strokes—one with a pontine infarction and one with an occipital lobe infarction. In the ≥70-year-old group, three patients (3.3%) suffered strokes in the target vessel territory, including one pontine infarction, one thalamic hemorrhage, and one midbrain infarction. There was no significant difference in the incidence of target vessel territory strokes between the two groups (Figure 2).

**FIGURE 3:**
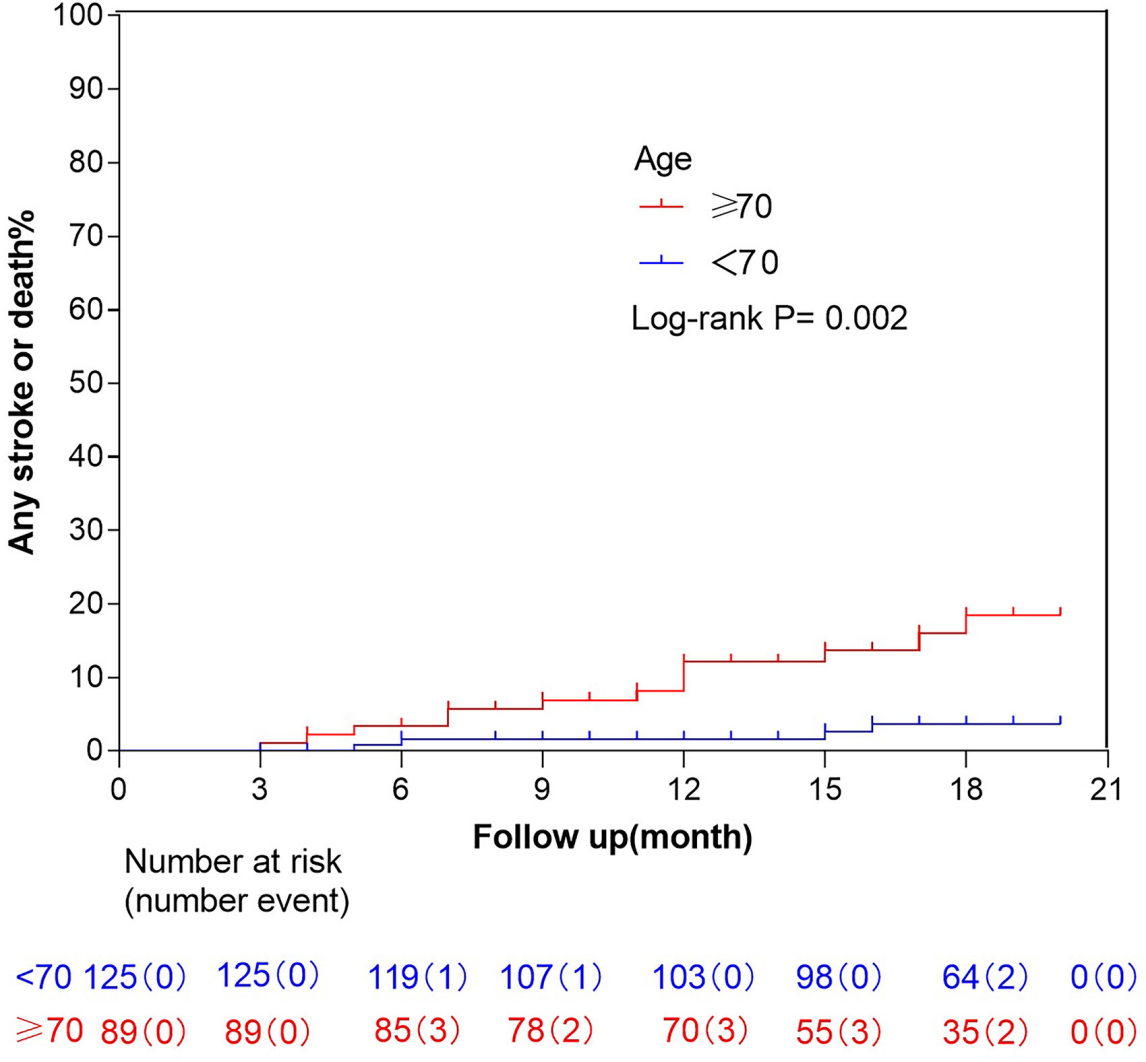
Kaplan-Meier curve of any stroke or death.

In the <70-year-old group, any stroke or death occurred in four patients (3.2%), including one pontine infarction, one occipital lobe infarction, one anterior circulation infarction, and one death due to frontal lobe hemorrhage following glioma surgery. In contrast, 13 patients (14.6%) in the ≥70-year-old group experienced stroke or death, comprising one pontine infarction, one thalamic hemorrhage, one midbrain infarction, seven minor anterior circulation strokes, one death due to traumatic intracranial hemorrhage, one pneumonia-related death, and one fatal massive middle cerebral artery embolism secondary to atrial fibrillation. The incidence of any stroke or death was significantly higher in the ≥70-year-old group compared to the <70-year-old group (Figure 3). Within the ≥ 70-year-old group, a sex-stratified analysis was performed for the occurrence of any stroke or death. Among 67 male patients, 13 (19.4%) experienced any stroke or death during follow-up, whereas 2 of 30 female patients (6.7%) had such events (*P* = 0.137). There was no significant association between sex and the occurrence of any stroke or death.

## 4 Discussion

There is currently no consensus on whether stent implantation or conservative medical therapy is more appropriate for patients with symptomatic vertebral artery stenosis. The SAMMPRIS (Derdeyn et al., 2014) trial demonstrated that for intracranial arterial stenosis, medical therapy alone was superior to stenting combined with medical therapy. The VAST (Compter et al., 2015) trial showed that the periprocedural risk of vascular events was higher in the stenting group than in the medical group. However, most adverse events in the stenting group occurred in patients receiving intracranial vertebral artery stenting, while those undergoing extracranial vertebral artery stenting had a lower incidence of periprocedural complications. In 2017, the VIST (Markus et al., 2017) study, which mainly focused on extracranial vertebral artery stenosis, indicated that the stenting group had fewer periprocedural complications compared to the medical group. Although no significant statistical difference was observed in the incidence of vertebrobasilar strokes during follow-up, a favorable trend toward stenting benefit was noted.

Moreover, a meta-analysis (Markus et al., 2019) combining data from the SAMMPRIS, VIST, and VAST trials suggested that stenting might benefit patients with extracranial vertebral artery stenosis. A review (Xu et al., 2022) based on these three studies also concluded that there was no significant difference in the short- or long-term risk of stroke, death, or transient ischemic attack (TIA) between patients receiving endovascular therapy plus medical treatment and those receiving medical treatment alone. Importantly, the anatomical location of the lesion—particularly the distinction between intracranial and extracranial stenosis—should not be overlooked in the management of symptomatic vertebral artery stenosis. An increasing number of studies have supported the safety and efficacy of stenting for symptomatic extracranial vertebral artery stenosis, especially at the vertebral artery origin. Currently, there is no evidence indicating that endovascular treatment is contraindicated in patients with medically refractory extracranial vertebral artery stenosis (El Koussa et al., 2022; Jing et al., 2025).

With the rise in life expectancy and the aging population, the incidence of vascular stenosis is increasing, and there is growing attention to endovascular therapy in the elderly (Halandras, 2019). To date, no studies have evaluated the safety and efficacy of stenting for symptomatic extracranial vertebral artery stenosis based on age stratification. This study found that the perioperative safety of vertebral artery stenting in symptomatic patients with vertebral artery stenosis aged ≥ 70 years was comparable to that of patients < 70 years. The rates of ISR and target vessel stroke recurrence were also similar between the two groups, with both groups exhibiting low event rates. Ma et al. (2021) included 101 patients who underwent rapamycin-eluting stent implantation in the vertebral artery, with a mean age of 62.87 ± 8.41 years. The perioperative stroke incidence was 0%. During an average follow-up of 12 months, the ISR rate was 5.9%, and the target vessel stroke recurrence rate was 2%, while the overall mortality rate was 6.1%. Li et al. (2020) included 137 patients who underwent vertebral artery stenting, including 76 patients with DES and 74 patients with BMS, with a mean age of 64 years. The perioperative adverse event rate in the DES group was 6.6%, and in the BMS group was 4.1%. During an average follow-up of 24 months, the ISR rate was 18.4% in the DES group and 31.1% in the BMS group, while the target vessel stroke recurrence rate was 8.7%. The results of these studies are consistent with our findings and align with previous research (Gupta et al., 2006; Langwieser et al., 2014; Maciejewski et al., 2019). Notably, the median age of the ≥70-year group in our study was 74 years, substantially higher than in previous studies, while the <70-year group had a median age of 63 years, comparable to past research. These findings demonstrate that stenting remains a safe and effective treatment option in patients aged ≥ 70 years.

The higher rate of any stroke in the ≥70-year group was mainly attributed to small infarcts in the anterior circulation. Except for one case of fatal massive cerebral embolism due to atrial fibrillation, no stroke with NIHSS > 4 was observed. Aging exacerbates vascular endothelial dysfunction, and atherosclerosis is a hallmark of vascular aging (Tyrrell et al., 2020). Oxidative stress and inflammation appear to be the two main underlying mechanisms (Tesauro et al., 2017; Watanabe and Hashimoto, 2022). Carotid atherosclerosis is regarded as a reflection of systemic atherosclerosis (Ihle-Hansen et al., 2023), and the increased incidence of anterior circulation small infarcts in older patients may be related to carotid artery disease. Studies have shown that carotid plaques increase the risk of cerebral infarction by 1.5 times. Severe carotid plaques significantly increase the risk of non-lacunar infarcts in the anterior circulation (RR 3.2 [95% CI, 1.1–9.7]) and lacunar infarcts (RR 10.8 [95% CI, 1.7–69.7]), but do not increase the risk in the posterior circulation (RR 0.6 [95% CI, 0.1–4.9]) (Hollander et al., 2002). Cerebral small vessel disease (CSVD), which becomes more prevalent with age, frequently affects the white matter and accounts for approximately 25% of ischemic strokes (Chojdak-Łukasiewicz et al., 2021). Both extracranial and intracranial atherosclerosis are associated with white matter hyperintensities seen in CSVD (Zhang et al., 2023), which may partly explain the high incidence of anterior circulation small strokes in the elderly. In our study, the all-cause mortality rate was higher in the elderly group, consistent with findings that age is an independent predictor of all-cause death (Sella et al., 2022). Other studies have suggested that age, comorbidities, modified Rankin Scale scores, and the number of vascular risk factors are associated with long-term outcomes after stroke in older adults. Although short-term functional outcomes may be similar between elderly and younger patients after adjusting for neurological deterioration, long-term survival in the elderly is often compromised by a higher burden of risk factors and comorbid complications such as pneumonia and cardiovascular diseases (Naess et al., 2014). Furthermore, as direct evidence regarding sex-related differences in stroke incidence after vertebral artery stenting remains limited in previous studies, we conducted a sex-stratified analysis among patients aged ≥ 70 years with symptomatic vertebral artery stenosis. No statistically significant difference was observed between males and females; however, male patients tended to have a higher risk of any stroke or death compared with females (19.4% vs. 6.7%). Given the relatively small sample size of our study, larger multicenter investigations are warranted to clarify whether sex serves as an independent risk factor for stroke and death following vertebral artery stenting.

In addition, we found that the increased risk of ISR was not associated with age, but was independently correlated with stent type and hyperuricemia. BMS were more prone to ISR than DES. The main difference between the two is that DES are coated either on the surface or inside with drugs that inhibit vascular endothelial cell proliferation. The continuous release of the drug from the stent can limit the proliferation and migration of smooth muscle cells in the stented vessel, thereby inhibiting in-stent thrombosis and preventing restenosis (Si et al., 2022). Several studies have shown that DES is superior to BMS in reducing ISR (Jia et al., 2022; Zhang et al., 2024), consistent with our findings. Serum uric acid, a byproduct of purine metabolism, has been reported to promote neoatherosclerosis within stents. This may occur through mechanisms involving the stimulation of inflammatory cytokines and reduced nitric oxide (NO) production, thereby impairing endothelial function (Gu et al., 2024). Other studies suggest that hyperuricemia may induce smooth muscle cell proliferation and increase the formation of thin-cap fibroatheromas, rendering plaques more vulnerable and increasing ISR risk (Zhao et al., 2017). ISR is a multifactorial complication influenced by sample size, follow-up duration, postoperative medication, technical factors, and surgical expertise.

This study has several limitations. First, this study is a single-center retrospective analysis, lacking randomization, and the patient selection for enrollment may have been subject to selection bias. Second, the average follow-up period of 15 months may be insufficient to fully assess ISR, target-vessel stroke, and other late events. Third, the sample size was relatively small, particularly in the ≥70-year group.

## 5 Conclusion

The use of extracranial vertebral artery stenting in elderly patients with symptomatic VAS has yielded favorable ISR outcomes and demonstrated a positive trend in clinical safety, including a low incidence of periprocedural complications and mid-term target-vessel territory strokes. Although the rate of any stroke or death was higher in patients aged ≥ 70 years, this was primarily attributed to underlying atherosclerosis rather than the procedure itself. Therefore, age ≥ 70 should not be considered a contraindication for stenting. This study provides an alternative treatment option for managing VAS in elderly patients. However, further prospective studies are needed to confirm the long-term clinical benefits of vertebral artery stenting in this population.

## Data Availability

The raw data supporting the conclusions of this article will be made available by the authors, without undue reservation.
